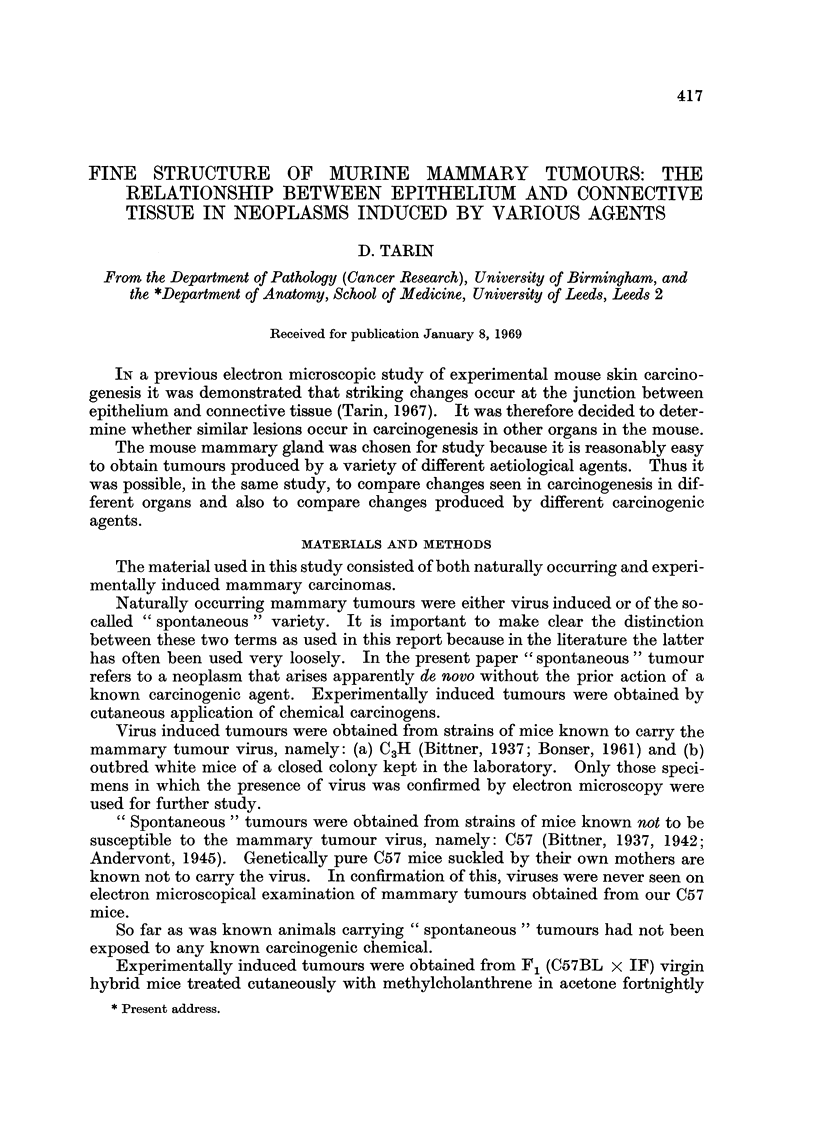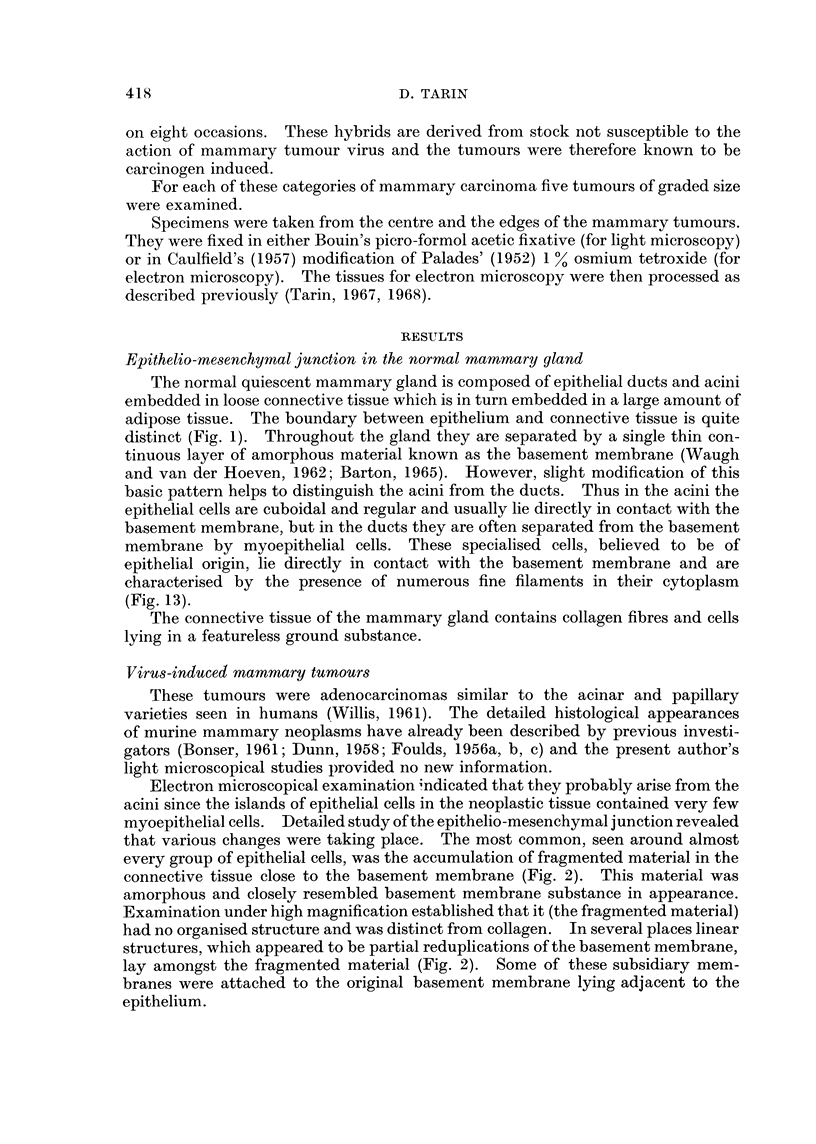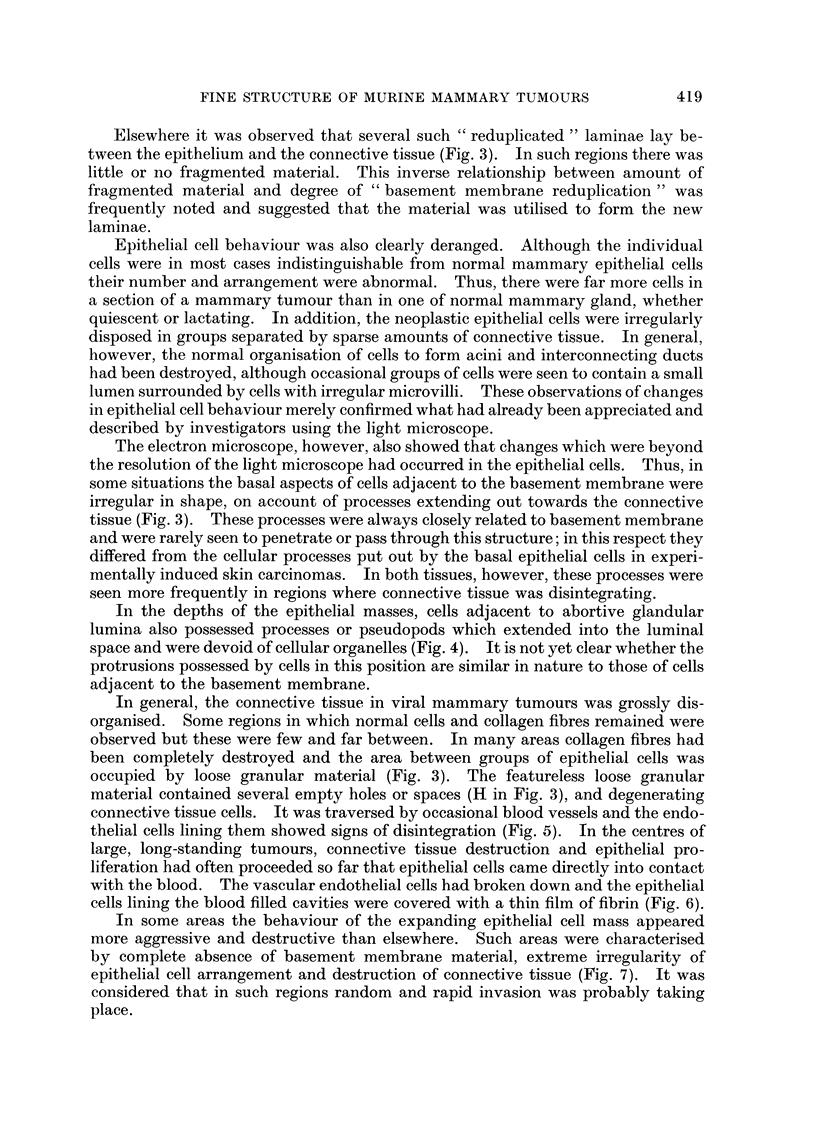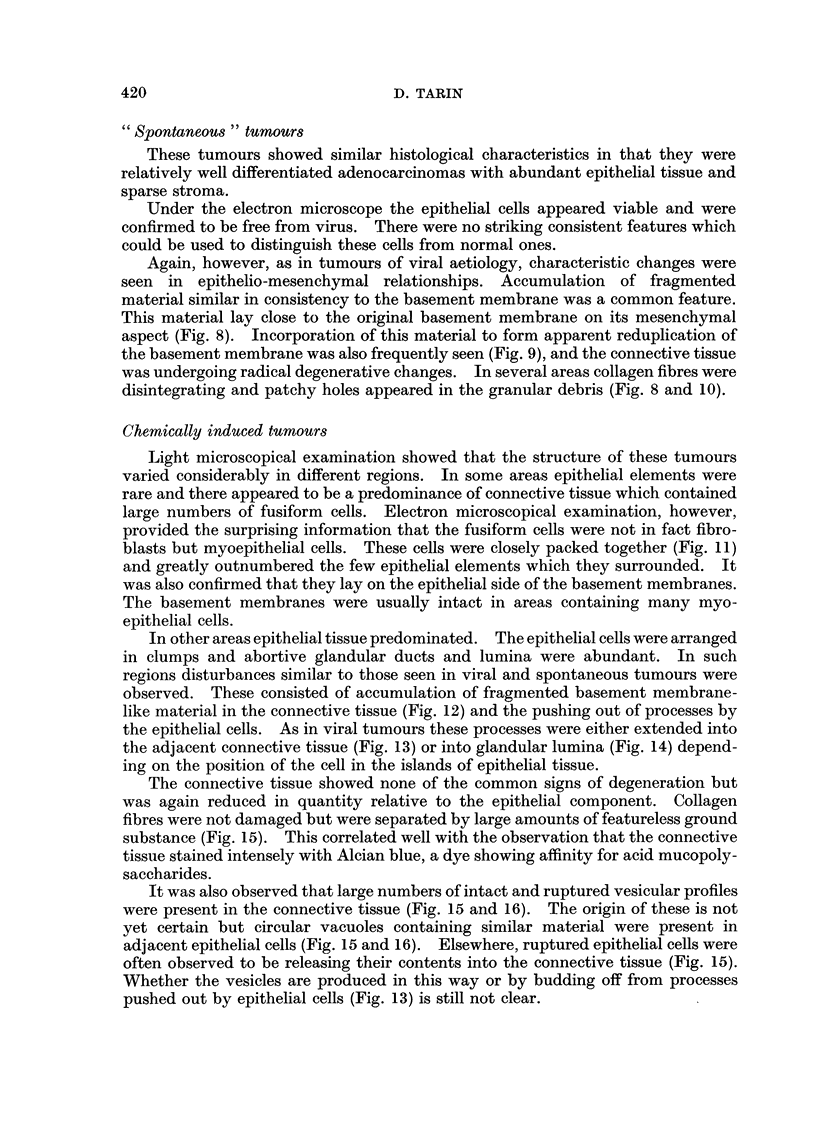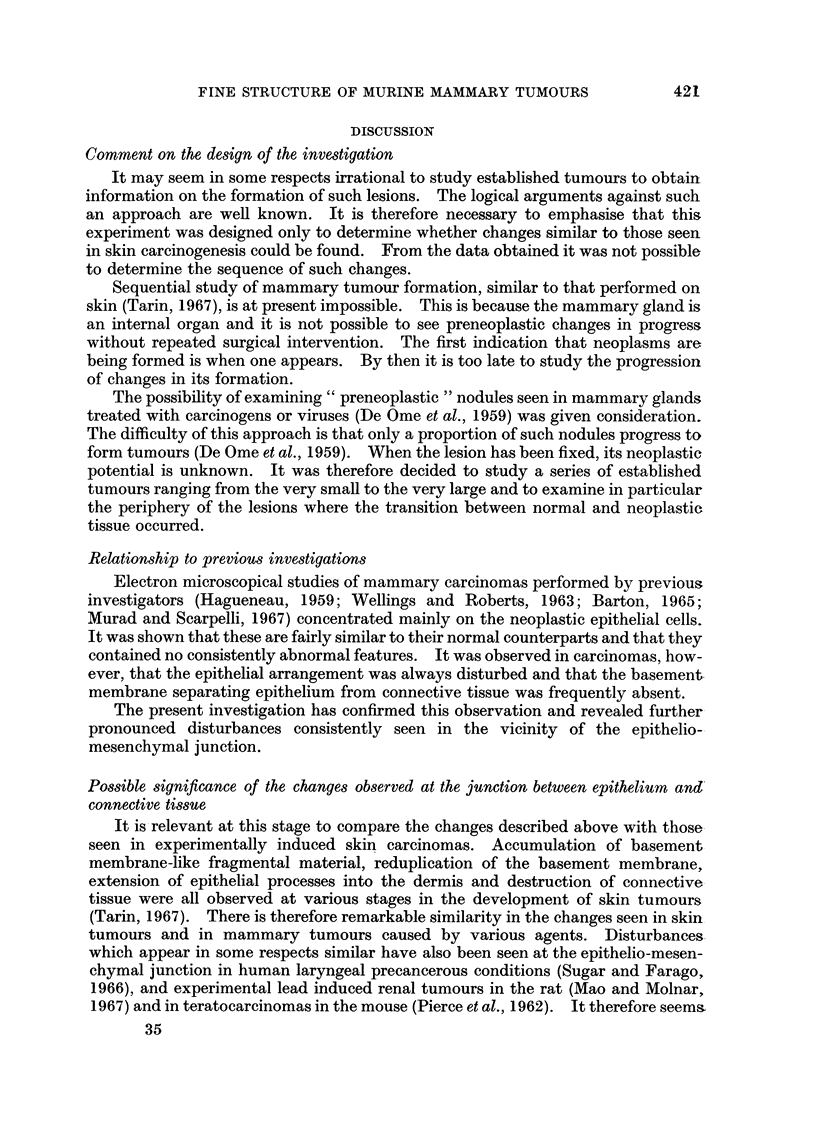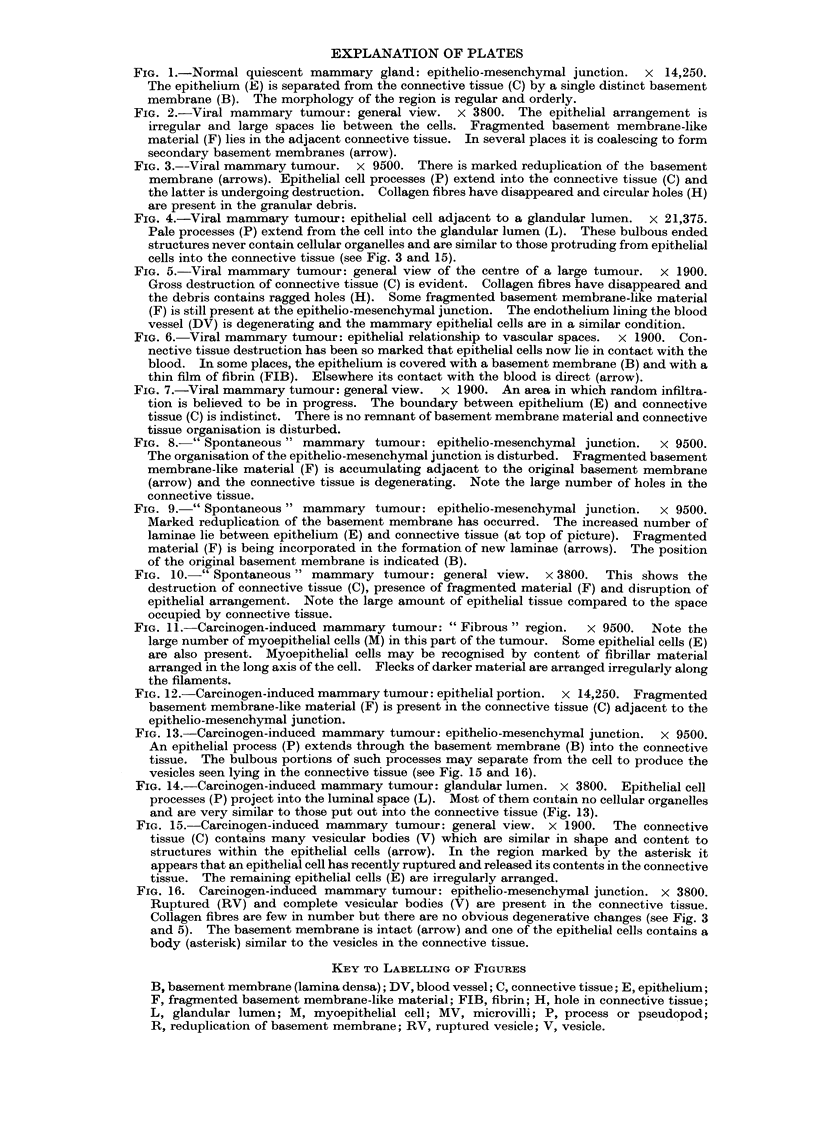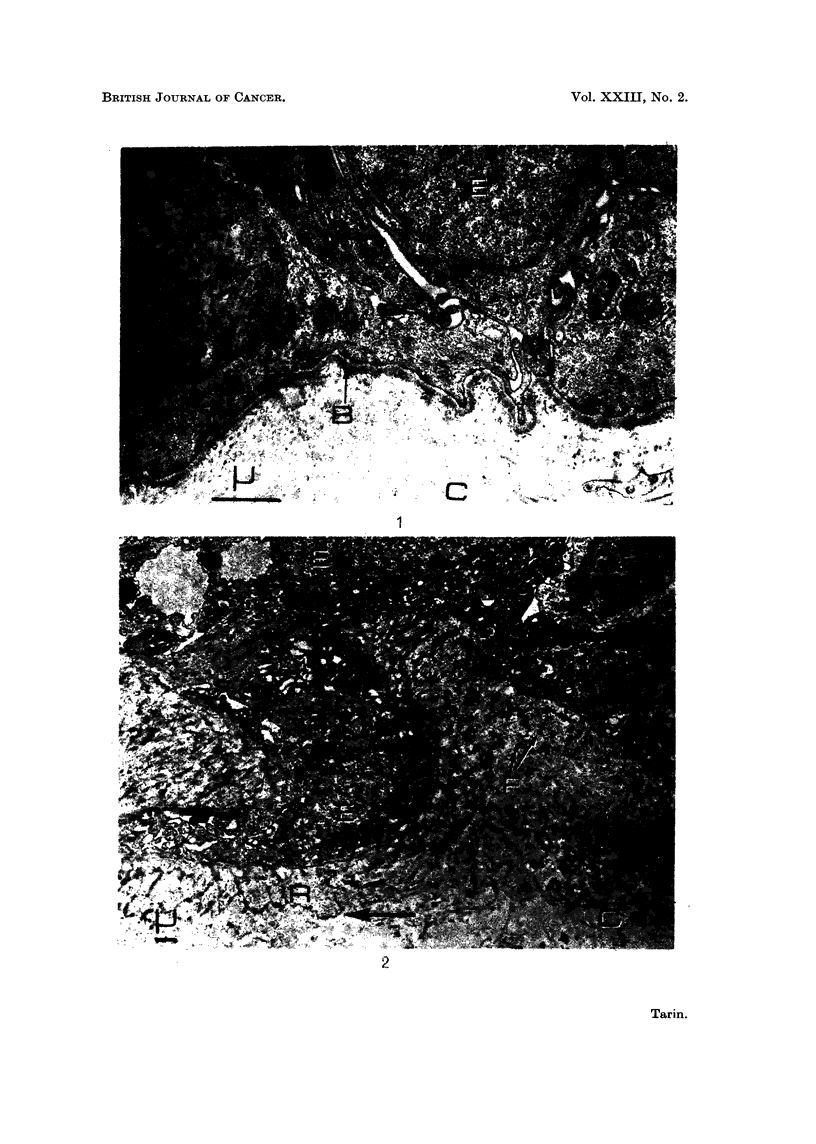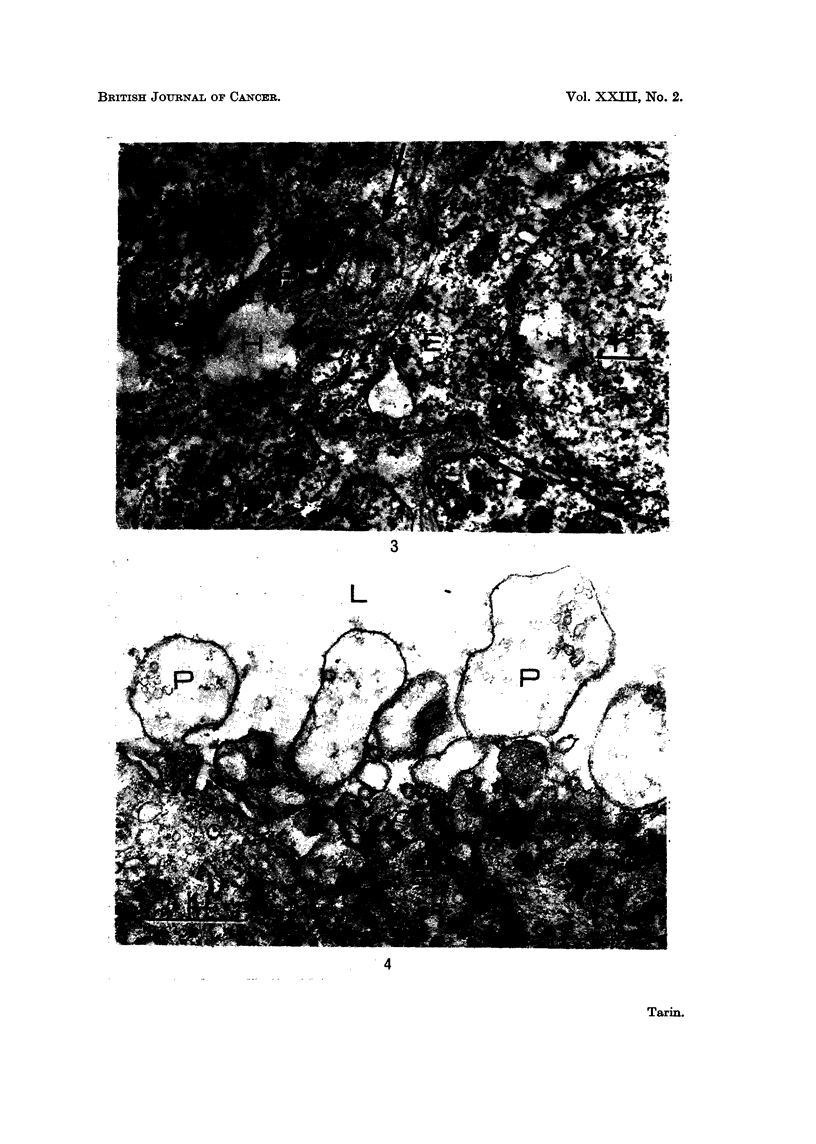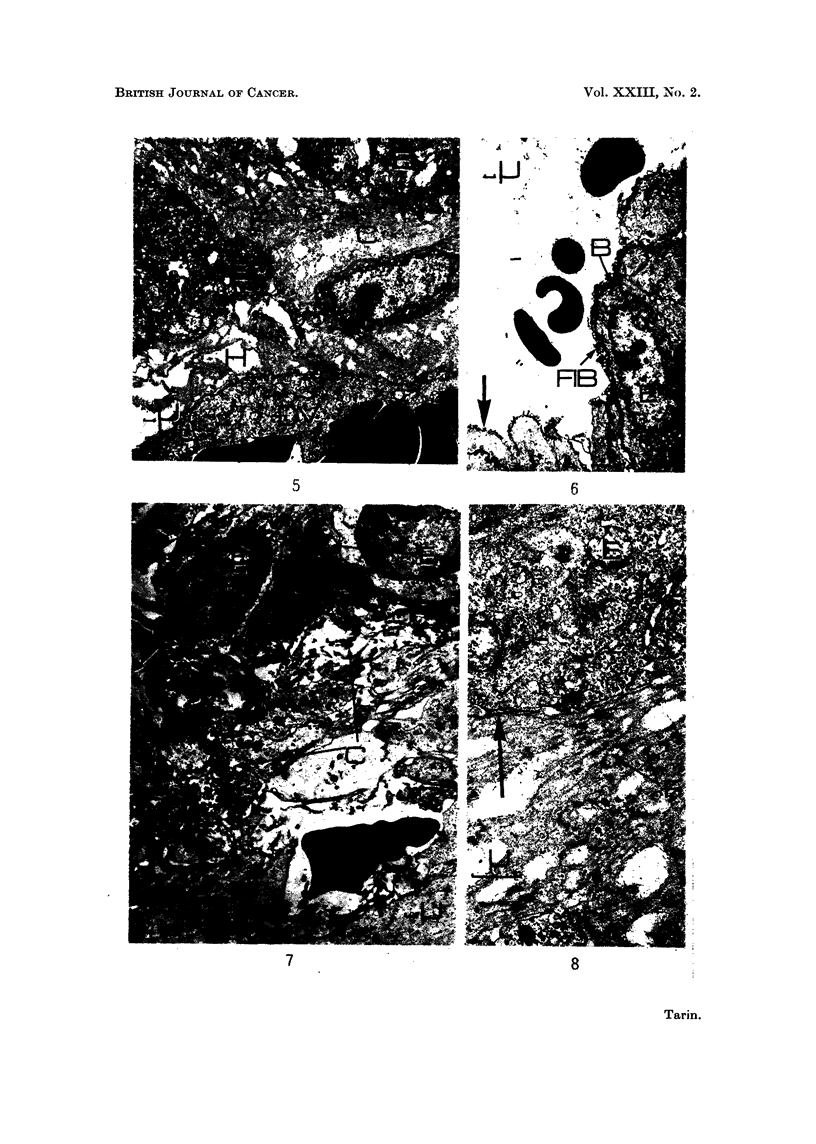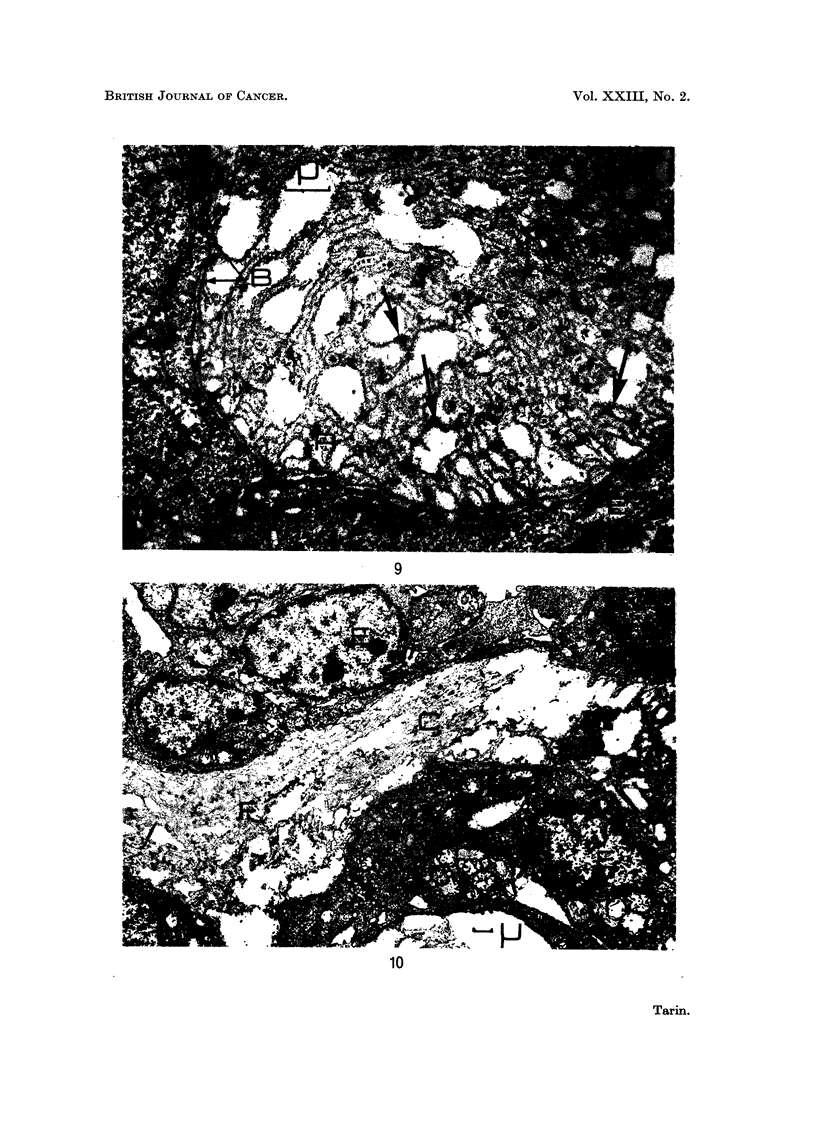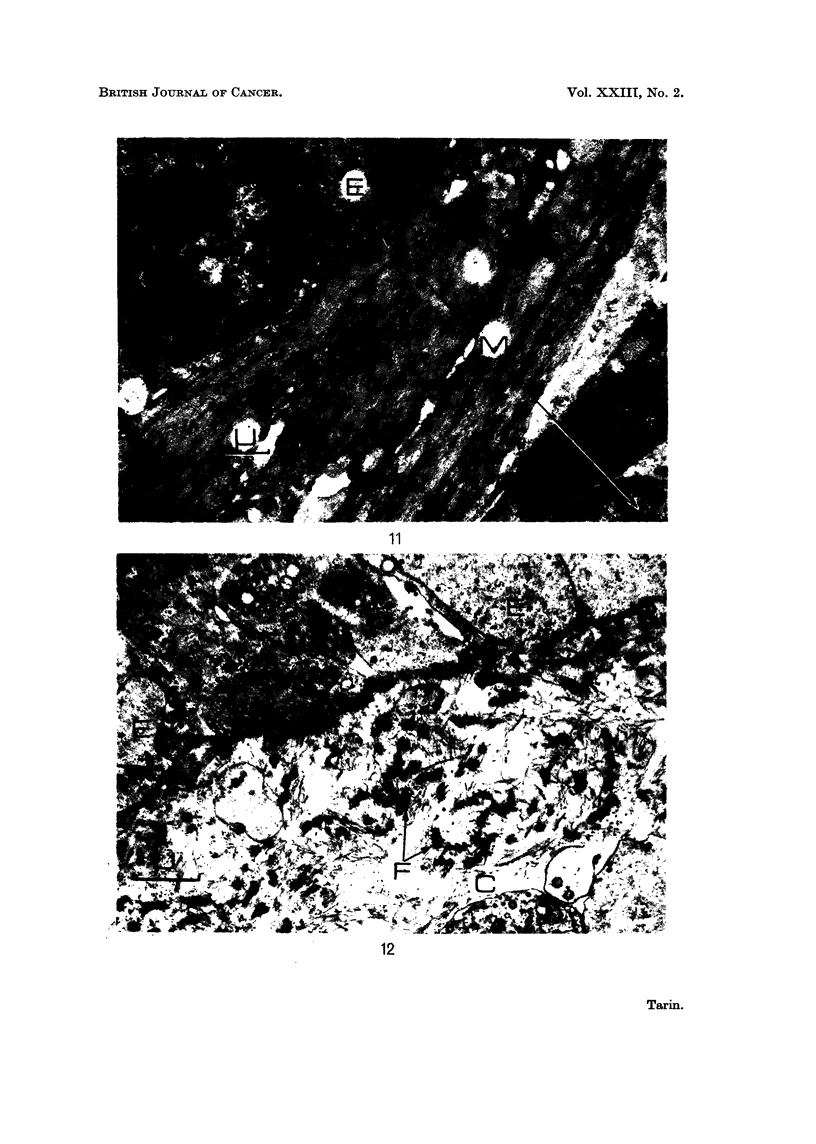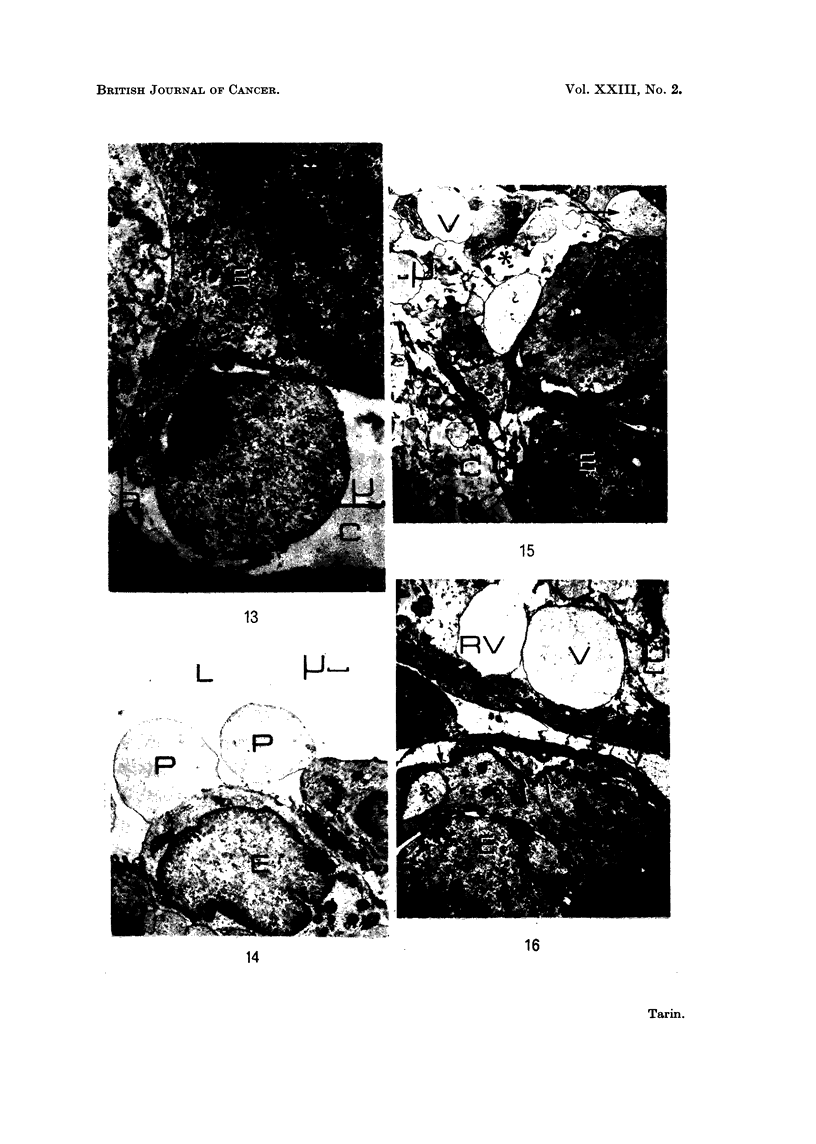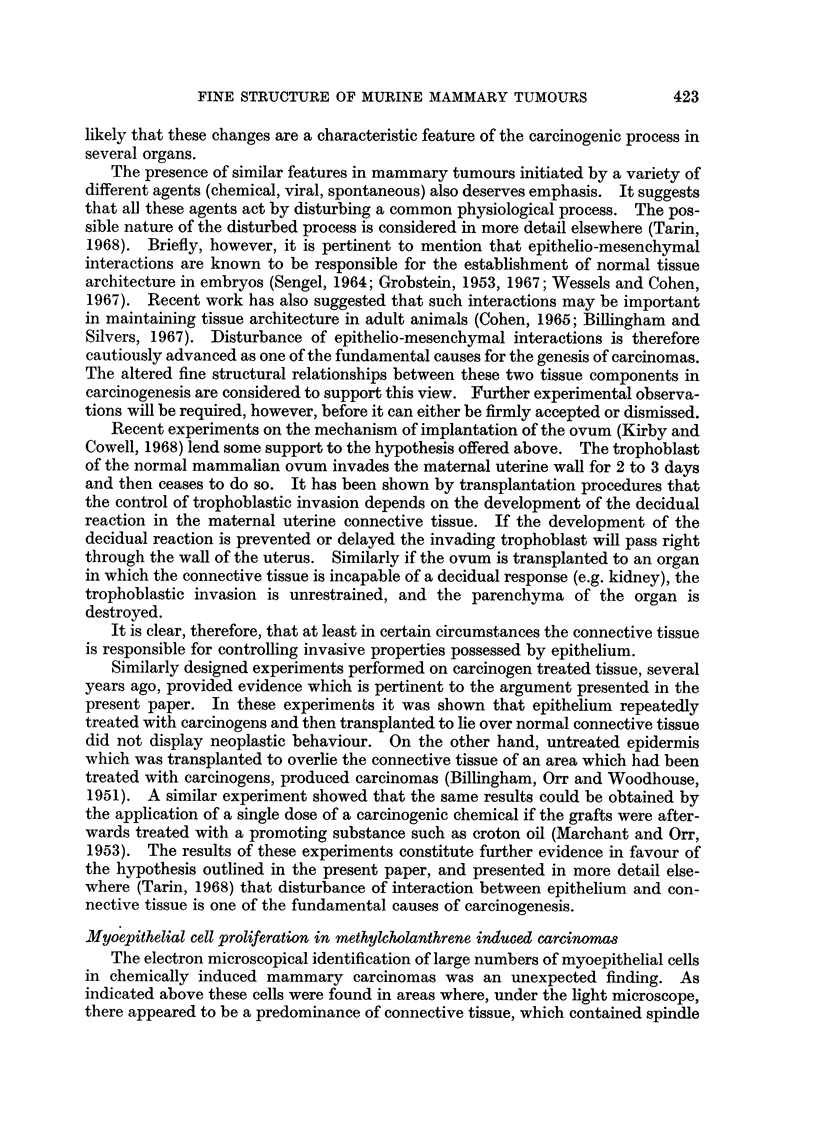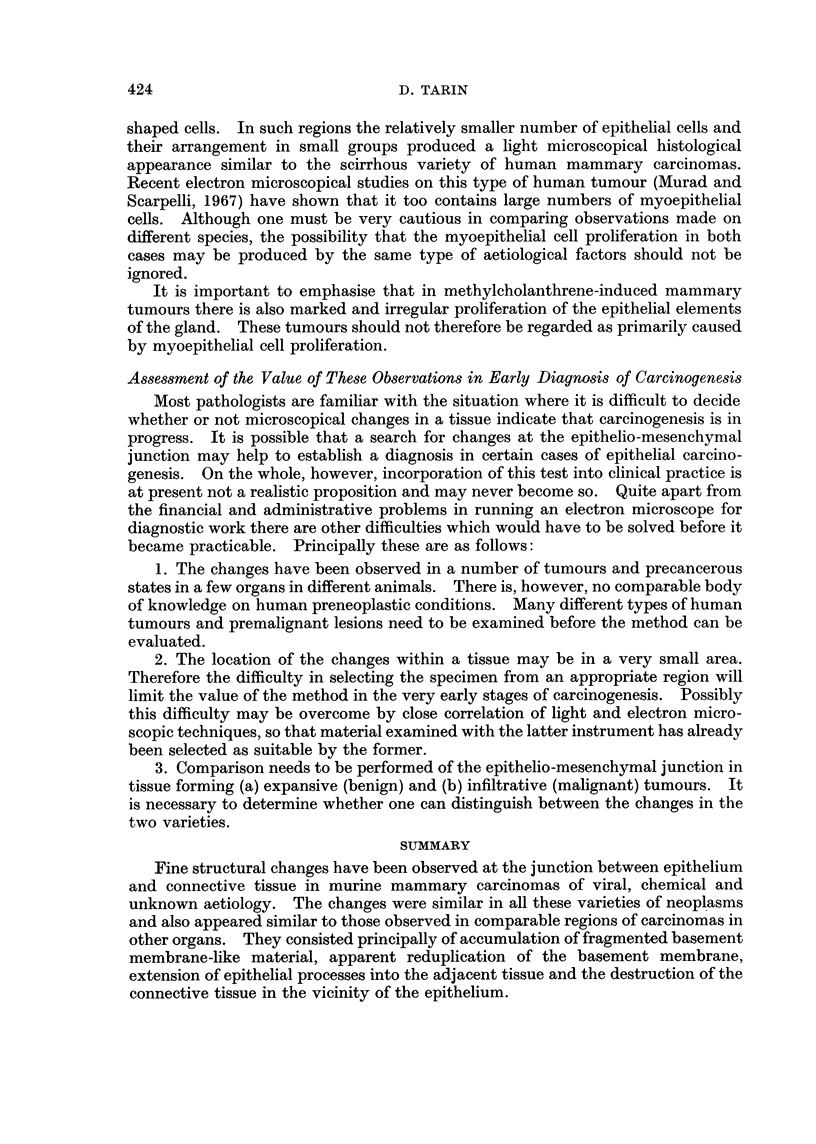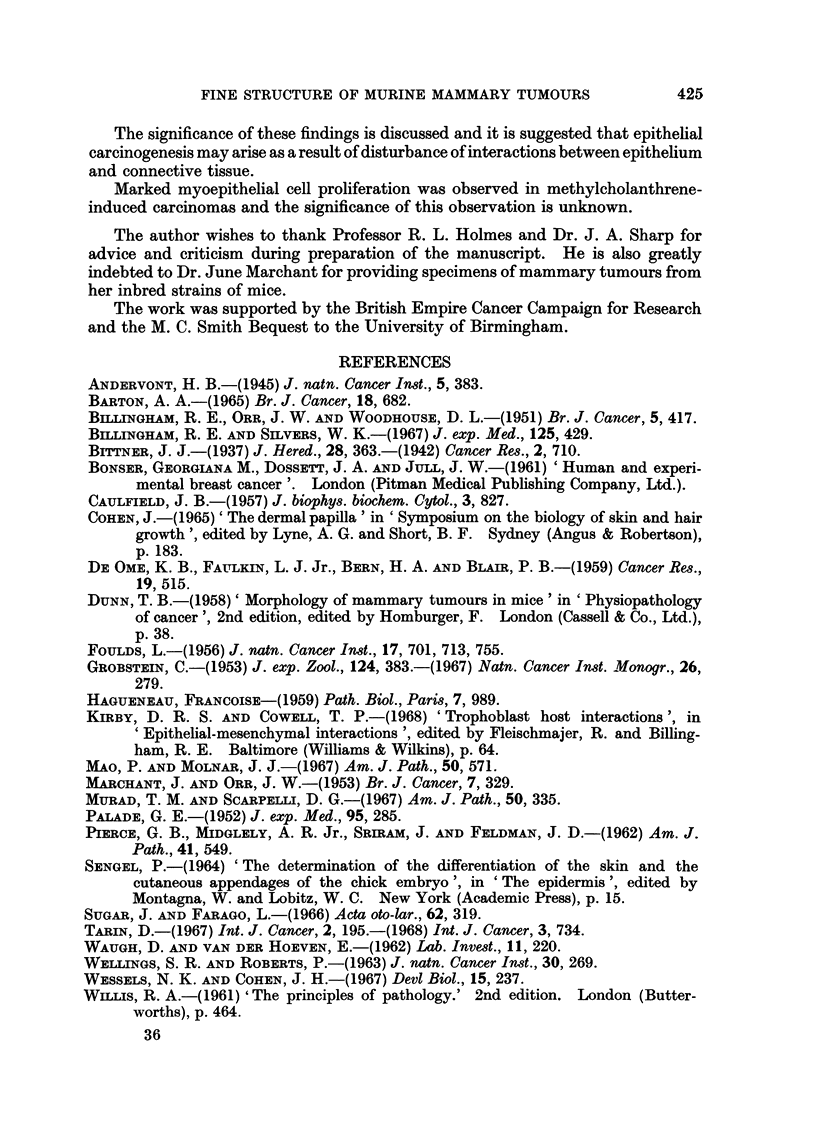# Fine structure of murine mammary tumours: the relationship between epithelium and connective tissue in neoplasms induced by various agents.

**DOI:** 10.1038/bjc.1969.54

**Published:** 1969-06

**Authors:** D. Tarin

## Abstract

**Images:**


### FINE STRUCTURE OF MURINE MAMMARY TUMOURS: THE RELATIONSHIP BETWEEN EPITHELIUM AND CONNECTIVE TISSUE IN NEOPLASMS INDUCED BY VARIOUS AGENTS

D. TARIN

*From the Department of Pathology (Cancer Research), University of Birmingham, and the *Department of Anatomy, School of Medicine, University of Leeds, Leeds 2*

Received for publication January 8, 1969

In a previous electron microscopic study of experimental mouse skin carcinogenesis it was demonstrated that striking changes occur at the junction between epithelium and connective tissue (Tarin, 1967). It was therefore decided to determine whether similar lesions occur in carcinogenesis in other organs in the mouse.

The mouse mammary gland was chosen for study because it is reasonably easy to obtain tumours produced by a variety of different aetiological agents. Thus it was possible, in the same study, to compare changes seen in carcinogenesis in different organs and also to compare changes produced by different carcinogenic agents.

### MATERIALS AND METHODS

The material used in this study consisted of both naturally occurring and experimentally induced mammary carcinomas.

Naturally occurring mammary tumours were either virus induced or of the so-called " spontaneous " variety. It is important to make clear the distinction between these two terms as used in this report because in the literature the latter has often been used very loosely. In the present paper " spontaneous " tumour refers to a neoplasm that arises apparently *de novo* without the prior action of a known carcinogenic agent. Experimentally induced tumours were obtained by cutaneous application of chemical carcinogens.

Virus induced tumours were obtained from strains of mice known to carry the mammary tumour virus, namely: (a) $C_3H$ (Bittner, 1937; Bonser, 1961) and (b) outbred white mice of a closed colony kept in the laboratory. Only those specimens in which the presence of virus was confirmed by electron microscopy were used for further study.

" Spontaneous " tumours were obtained from strains of mice known *not* to be susceptible to the mammary tumour virus, namely: C57 (Bittner, 1937, 1942; Andervont, 1945). Genetically pure C57 mice suckled by their own mothers are known not to carry the virus. In confirmation of this, viruses were never seen on electron microscopical examination of mammary tumours obtained from our C57 mice.

So far as was known animals carrying " spontaneous " tumours had not been exposed to any known carcinogenic chemical.

Experimentally induced tumours were obtained from $F_1$ (C57BL $\times$ IF) virgin hybrid mice treated cutaneously with methylcholanthrene in acetone fortnightly

* Present address.

on eight occasions. These hybrids are derived from stock not susceptible to the action of mammary tumour virus and the tumours were therefore known to be carcinogen induced.

For each of these categories of mammary carcinoma five tumours of graded size were examined.

Specimens were taken from the centre and the edges of the mammary tumours. They were fixed in either Bouin's picro-formol acetic fixative (for light microscopy) or in Caulfield's (1957) modification of Palades' (1952) 1 % osmium tetroxide (for electron microscopy). The tissues for electron microscopy were then processed as described previously (Tarin, 1967, 1968).

### RESULTS

### *Epithelio-mesenchymal junction in the normal mammary gland*

The normal quiescent mammary gland is composed of epithelial ducts and acini embedded in loose connective tissue which is in turn embedded in a large amount of adipose tissue. The boundary between epithelium and connective tissue is quite distinct (Fig. 1). Throughout the gland they are separated by a single thin continuous layer of amorphous material known as the basement membrane (Waugh and van der Hoeven, 1962; Barton, 1965). However, slight modification of this basic pattern helps to distinguish the acini from the ducts. Thus in the acini the epithelial cells are cuboidal and regular and usually lie directly in contact with the basement membrane, but in the ducts they are often separated from the basement membrane by myoepithelial cells. These specialised cells, believed to be of epithelial origin, lie directly in contact with the basement membrane and are characterised by the presence of numerous fine filaments in their cytoplasm (Fig. 13).

The connective tissue of the mammary gland contains collagen fibres and cells lying in a featureless ground substance.

### *Virus-induced mammary tumours*

These tumours were adenocarcinomas similar to the acinar and papillary varieties seen in humans (Willis, 1961). The detailed histological appearances of murine mammary neoplasms have already been described by previous investigators (Bonser, 1961; Dunn, 1958; Foulds, 1956a, b, c) and the present author's light microscopical studies provided no new information.

Electron microscopical examination indicated that they probably arise from the acini since the islands of epithelial cells in the neoplastic tissue contained very few myoepithelial cells. Detailed study of the epithelio-mesenchymal junction revealed that various changes were taking place. The most common, seen around almost every group of epithelial cells, was the accumulation of fragmented material in the connective tissue close to the basement membrane (Fig. 2). This material was amorphous and closely resembled basement membrane substance in appearance. Examination under high magnification established that it (the fragmented material) had no organised structure and was distinct from collagen. In several places linear structures, which appeared to be partial reduplications of the basement membrane, lay amongst the fragmented material (Fig. 2). Some of these subsidiary membranes were attached to the original basement membrane lying adjacent to the epithelium.

Elsewhere it was observed that several such " reduplicated " laminae lay between the epithelium and the connective tissue (Fig. 3). In such regions there was little or no fragmented material. This inverse relationship between amount of fragmented material and degree of " basement membrane reduplication " was frequently noted and suggested that the material was utilised to form the new laminae.

Epithelial cell behaviour was also clearly deranged. Although the individual cells were in most cases indistinguishable from normal mammary epithelial cells their number and arrangement were abnormal. Thus, there were far more cells in a section of a mammary tumour than in one of normal mammary gland, whether quiescent or lactating. In addition, the neoplastic epithelial cells were irregularly disposed in groups separated by sparse amounts of connective tissue. In general, however, the normal organisation of cells to form acini and interconnecting ducts had been destroyed, although occasional groups of cells were seen to contain a small lumen surrounded by cells with irregular microvilli. These observations of changes in epithelial cell behaviour merely confirmed what had already been appreciated and described by investigators using the light microscope.

The electron microscope, however, also showed that changes which were beyond the resolution of the light microscope had occurred in the epithelial cells. Thus, in some situations the basal aspects of cells adjacent to the basement membrane were irregular in shape, on account of processes extending out towards the connective tissue (Fig. 3). These processes were always closely related to basement membrane and were rarely seen to penetrate or pass through this structure; in this respect they differed from the cellular processes put out by the basal epithelial cells in experimentally induced skin carcinomas. In both tissues, however, these processes were seen more frequently in regions where connective tissue was disintegrating.

In the depths of the epithelial masses, cells adjacent to abortive glandular lumina also possessed processes or pseudopods which extended into the luminal space and were devoid of cellular organelles (Fig. 4). It is not yet clear whether the protrusions possessed by cells in this position are similar in nature to those of cells adjacent to the basement membrane.

In general, the connective tissue in viral mammary tumours was grossly disorganised. Some regions in which normal cells and collagen fibres remained were observed but these were few and far between. In many areas collagen fibres had been completely destroyed and the area between groups of epithelial cells was occupied by loose granular material (Fig. 3). The featureless loose granular material contained several empty holes or spaces (H in Fig. 3), and degenerating connective tissue cells. It was traversed by occasional blood vessels and the endothelial cells lining them showed signs of disintegration (Fig. 5). In the centres of large, long-standing tumours, connective tissue destruction and epithelial proliferation had often proceeded so far that epithelial cells came directly into contact with the blood. The vascular endothelial cells had broken down and the epithelial cells lining the blood filled cavities were covered with a thin film of fibrin (Fig. 6).

In some areas the behaviour of the expanding epithelial cell mass appeared more aggressive and destructive than elsewhere. Such areas were characterised by complete absence of basement membrane material, extreme irregularity of epithelial cell arrangement and destruction of connective tissue (Fig. 7). It was considered that in such regions random and rapid invasion was probably taking place.

*" Spontaneous " tumours*

These tumours showed similar histological characteristics in that they were relatively well differentiated adenocarcinomas with abundant epithelial tissue and sparse stroma.

Under the electron microscope the epithelial cells appeared viable and were confirmed to be free from virus. There were no striking consistent features which could be used to distinguish these cells from normal ones.

Again, however, as in tumours of viral aetiology, characteristic changes were seen in epithelio-mesenchymal relationships. Accumulation of fragmented material similar in consistency to the basement membrane was a common feature. This material lay close to the original basement membrane on its mesenchymal aspect (Fig. 8). Incorporation of this material to form apparent reduplication of the basement membrane was also frequently seen (Fig. 9), and the connective tissue was undergoing radical degenerative changes. In several areas collagen fibres were disintegrating and patchy holes appeared in the granular debris (Fig. 8 and 10).

*Chemically induced tumours*

Light microscopical examination showed that the structure of these tumours varied considerably in different regions. In some areas epithelial elements were rare and there appeared to be a predominance of connective tissue which contained large numbers of fusiform cells. Electron microscopical examination, however, provided the surprising information that the fusiform cells were not in fact fibroblasts but myoepithelial cells. These cells were closely packed together (Fig. 11) and greatly outnumbered the few epithelial elements which they surrounded. It was also confirmed that they lay on the epithelial side of the basement membranes. The basement membranes were usually intact in areas containing many myoepithelial cells.

In other areas epithelial tissue predominated. The epithelial cells were arranged in clumps and abortive glandular ducts and lumina were abundant. In such regions disturbances similar to those seen in viral and spontaneous tumours were observed. These consisted of accumulation of fragmented basement membrane-like material in the connective tissue (Fig. 12) and the pushing out of processes by the epithelial cells. As in viral tumours these processes were either extended into the adjacent connective tissue (Fig. 13) or into glandular lumina (Fig. 14) depending on the position of the cell in the islands of epithelial tissue.

The connective tissue showed none of the common signs of degeneration but was again reduced in quantity relative to the epithelial component. Collagen fibres were not damaged but were separated by large amounts of featureless ground substance (Fig. 15). This correlated well with the observation that the connective tissue stained intensely with Alcian blue, a dye showing affinity for acid mucopolysaccharides.

It was also observed that large numbers of intact and ruptured vesicular profiles were present in the connective tissue (Fig. 15 and 16). The origin of these is not yet certain but circular vacuoles containing similar material were present in adjacent epithelial cells (Fig. 15 and 16). Elsewhere, ruptured epithelial cells were often observed to be releasing their contents into the connective tissue (Fig. 15). Whether the vesicles are produced in this way or by budding off from processes pushed out by epithelial cells (Fig. 13) is still not clear.

### DISCUSSION

### *Comment on the design of the investigation*

It may seem in some respects irrational to study established tumours to obtain information on the formation of such lesions. The logical arguments against such an approach are well known. It is therefore necessary to emphasise that this experiment was designed only to determine whether changes similar to those seen in skin carcinogenesis could be found. From the data obtained it was not possible to determine the sequence of such changes.

Sequential study of mammary tumour formation, similar to that performed on skin (Tarin, 1967), is at present impossible. This is because the mammary gland is an internal organ and it is not possible to see preneoplastic changes in progress without repeated surgical intervention. The first indication that neoplasms are being formed is when one appears. By then it is too late to study the progression of changes in its formation.

The possibility of examining " preneoplastic " nodules seen in mammary glands treated with carcinogens or viruses (De Ome *et al.*, 1959) was given consideration. The difficulty of this approach is that only a proportion of such nodules progress to form tumours (De Ome *et al.*, 1959). When the lesion has been fixed, its neoplastic potential is unknown. It was therefore decided to study a series of established tumours ranging from the very small to the very large and to examine in particular the periphery of the lesions where the transition between normal and neoplastic tissue occurred.

### *Relationship to previous investigations*

Electron microscopical studies of mammary carcinomas performed by previous investigators (Hagueneau, 1959; Wellings and Roberts, 1963; Barton, 1965; Murad and Scarpelli, 1967) concentrated mainly on the neoplastic epithelial cells. It was shown that these are fairly similar to their normal counterparts and that they contained no consistently abnormal features. It was observed in carcinomas, however, that the epithelial arrangement was always disturbed and that the basement membrane separating epithelium from connective tissue was frequently absent.

The present investigation has confirmed this observation and revealed further pronounced disturbances consistently seen in the vicinity of the epithelio-mesenchymal junction.

### *Possible significance of the changes observed at the junction between epithelium and connective tissue*

It is relevant at this stage to compare the changes described above with those seen in experimentally induced skin carcinomas. Accumulation of basement membrane-like fragmental material, reduplication of the basement membrane, extension of epithelial processes into the dermis and destruction of connective tissue were all observed at various stages in the development of skin tumours (Tarin, 1967). There is therefore remarkable similarity in the changes seen in skin tumours and in mammary tumours caused by various agents. Disturbances which appear in some respects similar have also been seen at the epithelio-mesenchymal junction in human laryngeal precancerous conditions (Sugar and Farago, 1966), and experimental lead induced renal tumours in the rat (Mao and Molnar, 1967) and in teratocarcinomas in the mouse (Pierce *et al.*, 1962). It therefore seems

### EXPLANATION OF PLATES

FIG. 1.—Normal quiescent mammary gland: epithelio-mesenchymal junction. × 14,250. The epithelium (E) is separated from the connective tissue (C) by a single distinct basement membrane (B). The morphology of the region is regular and orderly.

FIG. 2.—Viral mammary tumour: general view. × 3800. The epithelial arrangement is irregular and large spaces lie between the cells. Fragmented basement membrane-like material (F) lies in the adjacent connective tissue. In several places it is coalescing to form secondary basement membranes (arrow).

FIG. 3.—Viral mammary tumour. × 9500. There is marked reduplication of the basement membrane (arrows). Epithelial cell processes (P) extend into the connective tissue (C) and the latter is undergoing destruction. Collagen fibres have disappeared and circular holes (H) are present in the granular debris.

FIG. 4.—Viral mammary tumour: epithelial cell adjacent to a glandular lumen. × 21,375. Pale processes (P) extend from the cell into the glandular lumen (L). These bulbous ended structures never contain cellular organelles and are similar to those protruding from epithelial cells into the connective tissue (see Fig. 3 and 15).

FIG. 5.—Viral mammary tumour: general view of the centre of a large tumour. × 1900. Gross destruction of connective tissue (C) is evident. Collagen fibres have disappeared and the debris contains ragged holes (H). Some fragmented basement membrane-like material (F) is still present at the epithelio-mesenchymal junction. The endothelium lining the blood vessel (DV) is degenerating and the mammary epithelial cells are in a similar condition.

FIG. 6.—Viral mammary tumour: epithelial relationship to vascular spaces. × 1900. Connective tissue destruction has been so marked that epithelial cells now lie in contact with the blood. In some places, the epithelium is covered with a basement membrane (B) and with a thin film of fibrin (FIB). Elsewhere its contact with the blood is direct (arrow).

FIG. 7.—Viral mammary tumour: general view. × 1900. An area in which random infiltration is believed to be in progress. The boundary between epithelium (E) and connective tissue (C) is indistinct. There is no remnant of basement membrane material and connective tissue organisation is disturbed.

FIG. 8.—" Spontaneous " mammary tumour: epithelio-mesenchymal junction. × 9500. The organisation of the epithelio-mesenchymal junction is disturbed. Fragmented basement membrane-like material (F) is accumulating adjacent to the original basement membrane (arrow) and the connective tissue is degenerating. Note the large number of holes in the connective tissue.

FIG. 9.—" Spontaneous " mammary tumour: epithelio-mesenchymal junction. × 9500. Marked reduplication of the basement membrane has occurred. The increased number of laminae lie between epithelium (E) and connective tissue (at top of picture). Fragmented material (F) is being incorporated in the formation of new laminae (arrows). The position of the original basement membrane is indicated (B).

FIG. 10.—" Spontaneous " mammary tumour: general view. ×3800. This shows the destruction of connective tissue (C), presence of fragmented material (F) and disruption of epithelial arrangement. Note the large amount of epithelial tissue compared to the space occupied by connective tissue.

FIG. 11.—Carcinogen-induced mammary tumour: " Fibrous " region. × 9500. Note the large number of myoepithelial cells (M) in this part of the tumour. Some epithelial cells (E) are also present. Myoepithelial cells may be recognised by content of fibrillar material arranged in the long axis of the cell. Flecks of darker material are arranged irregularly along the filaments.

FIG. 12.—Carcinogen-induced mammary tumour: epithelial portion. × 14,250. Fragmented basement membrane-like material (F) is present in the connective tissue (C) adjacent to the epithelio-mesenchymal junction.

FIG. 13.—Carcinogen-induced mammary tumour: epithelio-mesenchymal junction. × 9500. An epithelial process (P) extends through the basement membrane (B) into the connective tissue. The bulbous portions of such processes may separate from the cell to produce the vesicles seen lying in the connective tissue (see Fig. 15 and 16).

FIG. 14.—Carcinogen-induced mammary tumour: glandular lumen. × 3800. Epithelial cell processes (P) project into the luminal space (L). Most of them contain no cellular organelles and are very similar to those put out into the connective tissue (Fig. 13).

FIG. 15.—Carcinogen-induced mammary tumour: general view. × 1900. The connective tissue (C) contains many vesicular bodies (V) which are similar in shape and content to structures within the epithelial cells (arrow). In the region marked by the asterisk it appears that an epithelial cell has recently ruptured and released its contents in the connective tissue. The remaining epithelial cells (E) are irregularly arranged.

FIG. 16. Carcinogen-induced mammary tumour: epithelio-mesenchymal junction. × 3800. Ruptured (RV) and complete vesicular bodies (V) are present in the connective tissue. Collagen fibres are few in number but there are no obvious degenerative changes (see Fig. 3 and 5). The basement membrane is intact (arrow) and one of the epithelial cells contains a body (asterisk) similar to the vesicles in the connective tissue.

### KEY TO LABELLING OF FIGURES

B, basement membrane (lamina densa); DV, blood vessel; C, connective tissue; E, epithelium; F, fragmented basement membrane-like material; FIB, fibrin; H, hole in connective tissue; L, glandular lumen; M, myoepithelial cell; MV, microvilli; P, process or pseudopod; R, reduplication of basement membrane; RV, ruptured vesicle; V, vesicle.

1

2

3

4

5

6

7

8

Tarin.

9

10

11

12

Tarin.

13 14 15 16

likely that these changes are a characteristic feature of the carcinogenic process in several organs.

The presence of similar features in mammary tumours initiated by a variety of different agents (chemical, viral, spontaneous) also deserves emphasis. It suggests that all these agents act by disturbing a common physiological process. The possible nature of the disturbed process is considered in more detail elsewhere (Tarin, 1968). Briefly, however, it is pertinent to mention that epithelio-mesenchymal interactions are known to be responsible for the establishment of normal tissue architecture in embryos (Sengel, 1964; Grobstein, 1953, 1967; Wessels and Cohen, 1967). Recent work has also suggested that such interactions may be important in maintaining tissue architecture in adult animals (Cohen, 1965; Billingham and Silvers, 1967). Disturbance of epithelio-mesenchymal interactions is therefore cautiously advanced as one of the fundamental causes for the genesis of carcinomas. The altered fine structural relationships between these two tissue components in carcinogenesis are considered to support this view. Further experimental observations will be required, however, before it can either be firmly accepted or dismissed.

Recent experiments on the mechanism of implantation of the ovum (Kirby and Cowell, 1968) lend some support to the hypothesis offered above. The trophoblast of the normal mammalian ovum invades the maternal uterine wall for 2 to 3 days and then ceases to do so. It has been shown by transplantation procedures that the control of trophoblastic invasion depends on the development of the decidual reaction in the maternal uterine connective tissue. If the development of the decidual reaction is prevented or delayed the invading trophoblast will pass right through the wall of the uterus. Similarly if the ovum is transplanted to an organ in which the connective tissue is incapable of a decidual response (e.g. kidney), the trophoblastic invasion is unrestrained, and the parenchyma of the organ is destroyed.

It is clear, therefore, that at least in certain circumstances the connective tissue is responsible for controlling invasive properties possessed by epithelium.

Similarly designed experiments performed on carcinogen treated tissue, several years ago, provided evidence which is pertinent to the argument presented in the present paper. In these experiments it was shown that epithelium repeatedly treated with carcinogens and then transplanted to lie over normal connective tissue did not display neoplastic behaviour. On the other hand, untreated epidermis which was transplanted to overlie the connective tissue of an area which had been treated with carcinogens, produced carcinomas (Billingham, Orr and Woodhouse, 1951). A similar experiment showed that the same results could be obtained by the application of a single dose of a carcinogenic chemical if the grafts were afterwards treated with a promoting substance such as croton oil (Marchant and Orr, 1953). The results of these experiments constitute further evidence in favour of the hypothesis outlined in the present paper, and presented in more detail elsewhere (Tarin, 1968) that disturbance of interaction between epithelium and connective tissue is one of the fundamental causes of carcinogenesis.

*Myoepithelial cell proliferation in methylcholanthrene induced carcinomas*

The electron microscopical identification of large numbers of myoepithelial cells in chemically induced mammary carcinomas was an unexpected finding. As indicated above these cells were found in areas where, under the light microscope, there appeared to be a predominance of connective tissue, which contained spindle shaped cells. In such regions the relatively smaller number of epithelial cells and their arrangement in small groups produced a light microscopical histological appearance similar to the scirrhous variety of human mammary carcinomas. Recent electron microscopical studies on this type of human tumour (Murad and Scarpelli, 1967) have shown that it too contains large numbers of myoepithelial cells. Although one must be very cautious in comparing observations made on different species, the possibility that the myoepithelial cell proliferation in both cases may be produced by the same type of aetiological factors should not be ignored.

It is important to emphasise that in methylcholanthrene-induced mammary tumours there is also marked and irregular proliferation of the epithelial elements of the gland. These tumours should not therefore be regarded as primarily caused by myoepithelial cell proliferation.

*Assessment of the Value of These Observations in Early Diagnosis of Carcinogenesis*

Most pathologists are familiar with the situation where it is difficult to decide whether or not microscopical changes in a tissue indicate that carcinogenesis is in progress. It is possible that a search for changes at the epithelio-mesenchymal junction may help to establish a diagnosis in certain cases of epithelial carcinogenesis. On the whole, however, incorporation of this test into clinical practice is at present not a realistic proposition and may never become so. Quite apart from the financial and administrative problems in running an electron microscope for diagnostic work there are other difficulties which would have to be solved before it became practicable. Principally these are as follows:

1. The changes have been observed in a number of tumours and precancerous states in a few organs in different animals. There is, however, no comparable body of knowledge on human preneoplastic conditions. Many different types of human tumours and premalignant lesions need to be examined before the method can be evaluated.

2. The location of the changes within a tissue may be in a very small area. Therefore the difficulty in selecting the specimen from an appropriate region will limit the value of the method in the very early stages of carcinogenesis. Possibly this difficulty may be overcome by close correlation of light and electron microscopic techniques, so that material examined with the latter instrument has already been selected as suitable by the former.

3. Comparison needs to be performed of the epithelio-mesenchymal junction in tissue forming (a) expansive (benign) and (b) infiltrative (malignant) tumours. It is necessary to determine whether one can distinguish between the changes in the two varieties.

SUMMARY

Fine structural changes have been observed at the junction between epithelium and connective tissue in murine mammary carcinomas of viral, chemical and unknown aetiology. The changes were similar in all these varieties of neoplasms and also appeared similar to those observed in comparable regions of carcinomas in other organs. They consisted principally of accumulation of fragmented basement membrane-like material, apparent reduplication of the basement membrane, extension of epithelial processes into the adjacent tissue and the destruction of the connective tissue in the vicinity of the epithelium.

The significance of these findings is discussed and it is suggested that epithelial carcinogenesis may arise as a result of disturbance of interactions between epithelium and connective tissue.

Marked myoepithelial cell proliferation was observed in methylcholanthrene-induced carcinomas and the significance of this observation is unknown.

The author wishes to thank Professor R. L. Holmes and Dr. J. A. Sharp for advice and criticism during preparation of the manuscript. He is also greatly indebted to Dr. June Marchant for providing specimens of mammary tumours from her inbred strains of mice.

The work was supported by the British Empire Cancer Campaign for Research and the M. C. Smith Bequest to the University of Birmingham.

### REFERENCES

ANDERVONT, H. B.—(1945) *J. natn. Cancer Inst.*, **5**, 383.
BARTON, A. A.—(1965) *Br. J. Cancer*, **18**, 682.
BILLINGHAM, R. E., ORR, J. W. AND WOODHOUSE, D. L.—(1951) *Br. J. Cancer*, **5**, 417.
BILLINGHAM, R. E. AND SILVERS, W. K.—(1967) *J. exp. Med.*, **125**, 429.
BITTNER, J. J.—(1937) *J. Hered.*, **28**, 363.—(1942) *Cancer Res.*, **2**, 710.
BONSER, GEORGIANA M., DOSSETT, J. A. AND JULL, J. W.—(1961) ' Human and experimental breast cancer '. London (Pitman Medical Publishing Company, Ltd.).
CAULFIELD, J. B.—(1957) *J. biophys. biochem. Cytol.*, **3**, 827.
COHEN, J.—(1965) ' The dermal papilla ' in ' Symposium on the biology of skin and hair growth ', edited by Lyne, A. G. and Short, B. F. Sydney (Angus & Robertson), p. 183.
DE OME, K. B., FAULKIN, L. J. Jr., BERN, H. A. AND BLAIR, P. B.—(1959) *Cancer Res.*, **19**, 515.
DUNN, T. B.—(1958) ' Morphology of mammary tumours in mice ' in ' Physiopathology of cancer ', 2nd edition, edited by Homburger, F. London (Cassell & Co., Ltd.), p. 38.
FOULDS, L.—(1956) *J. natn. Cancer Inst.*, **17**, 701, 713, 755.
GROBSTEIN, C.—(1953) *J. exp. Zool.*, **124**, 383.—(1967) *Natn. Cancer Inst. Monogr.*, **26**, 279.
HAGUENEAU, FRANCOISE—(1959) *Path. Biol., Paris*, **7**, 989.
KIRBY, D. R. S. AND COWELL, T. P.—(1968) ' Trophoblast host interactions ', in ' Epithelial-mesenchymal interactions ', edited by Fleischmajer, R. and Billingham, R. E. Baltimore (Williams & Wilkins), p. 64.
MAO, P. AND MOLNAR, J. J.—(1967) *Am. J. Path.*, **50**, 571.
MARCHANT, J. AND ORR, J. W.—(1953) *Br. J. Cancer*, **7**, 329.
MURAD, T. M. AND SCARPELLI, D. G.—(1967) *Am. J. Path.*, **50**, 335.
PALADE, G. E.—(1952) *J. exp. Med.*, **95**, 285.
PIERCE, G. B., MIDGLELY, A. R. Jr., SRIRAM, J. AND FELDMAN, J. D.—(1962) *Am. J. Path.*, **41**, 549.
SENGEL, P.—(1964) ' The determination of the differentiation of the skin and the cutaneous appendages of the chick embryo ', in ' The epidermis ', edited by Montagna, W. and Lobitz, W. C. New York (Academic Press), p. 15.
SUGAR, J. AND FARAGO, L.—(1966) *Acta oto-lar.*, **62**, 319.
TARIN, D.—(1967) *Int. J. Cancer*, **2**, 195.—(1968) *Int. J. Cancer*, **3**, 734.
WAUGH, D. AND VAN DER HOEVEN, E.—(1962) *Lab. Invest.*, **11**, 220.
WELLINGS, S. R. AND ROBERTS, P.—(1963) *J. natn. Cancer Inst.*, **30**, 269.
WESSELS, N. K. AND COHEN, J. H.—(1967) *Devl Biol.*, **15**, 237.
WILLIS, R. A.—(1961) 'The principles of pathology.' 2nd edition. London (Butterworths), p. 464.